# Assessment of Clinician Well-Being Using a Biometric-Informed Coaching Platform

**DOI:** 10.1001/jamanetworkopen.2025.58865

**Published:** 2026-02-11

**Authors:** Troy Leo, James Reynolds, Joshua Blair, Allyn Abadie, Elizabeth Euiler, Kevin Aubol, Wayne Sotile, Stephanie O’Bryon, Jeremiah Gaddy, Kevin W. Lobdell, Geoffrey A. Rose

**Affiliations:** 1 Sanger Heart & Vascular Institute, Advocate Health, Charlotte, North Carolina; 2 Arena Labs, Nashville, Tennessee; 3 Sotile Center for Resilience, Davidson, North Carolina; 4 Department of Emergency Medicine, Advocate Health, Charlotte, North Carolina

## Abstract

**Importance:**

The demands of clinical practice have contributed to widespread burnout among health care professionals. Thus, there is an urgent need for scalable and evidence-based interventions to address burnout in this population.

**Objective:**

To assess whether participation in an asynchronous, app-based, personalized coaching intervention informed by biometric data is associated with changes in burnout, professional fulfillment, and self-valuation among health care professionals.

**Design, Setting, and Participants:**

This prospective cohort study was conducted at a large health care system (Advocate Health) in the US. Clinicians and other health care professionals from 4 service lines (critical care, women’s health, heart and vascular, and emergency medicine) were recruited between June 14 and August 12, 2024. Between August 27 and October 22, 2024, participants completed assessments at baseline and 8 weeks. Those who completed assessments at both baseline and 8 weeks were included in this study. Analysis was completed in November 2024 and April 2025.

**Exposure:**

An 8-week asynchronous app-based coaching program integrated with a wearable photoplethysmography sensor.

**Main Outcomes and Measures:**

The primary outcome assessed was the change from baseline to week 8 in scores on Stanford Professional Fulfillment Index (PFI) domains (burnout, professional fulfillment, and self-valuation) and on Maslach Burnout Inventory Human Services Survey (MBI-HSS) subscales (emotional exhaustion, depersonalization, and personal accomplishment). For each outcome, linear mixed-effects models were used with restricted maximum likelihood.

**Results:**

A total of 367 clinicians and other health care professionals completed baseline assessments. Of these, 192 (52.3%) completed assessments at both baseline and 8 weeks and were included in this study. Their mean (SD) age was 42.5 (10.0) years, and most (135 [70.3%]) were female. The largest participant groups were attending physicians (92 [47.9%]), advanced practice clinicians (47 [24.5%]), and nurses (37 [19.3%]). For the PFI domains, the mean (SD) professional fulfillment score increased from 2.36 (0.68) to 2.56 (0.76) (change, 0.20 [95% CI, 0.12 to 0.28] points; *P* < .001), the mean (SD) burnout score decreased from 1.60 (0.71) to 1.09 (0.62) (change, −0.51 [95% CI, −0.60 to −0.43] points; *P* < .001), and the mean (SD) self-valuation score increased from 6.47 (3.00) to 9.02 (2.75) (change, 2.55 [95% CI, 2.12 to 2.98] points; *P* < .001). For the MBI-HSS subscales, the mean (SD) emotional exhaustion score decreased from 19.74 (8.95) to 15.68 (8.59) (change, −4.06 [95% CI, −5.10 to −3.03] points; *P* < .001), the mean (SD) depersonalization score decreased from 14.58 (8.50) to 10.32 (7.19) (change, −4.26 [95% CI, −5.26 to −3.27] points; *P* < .001), and the mean (SD) personal accomplishment score increased from 37.68 (6.85) to 39.19 (6.80) (change, 1.51 [95% CI, 0.75 to 2.28] points; *P* < .001).

**Conclusions and Relevance:**

In this cohort study of clinicians and other health care professionals, an asynchronous, biometric-informed coaching platform was associated with meaningful reductions in burnout and improvements in professional fulfillment and self-valuation. These findings suggest that adopting such a platform may be a feasible approach to support health care workers’ well-being.

## Key Points

**Question:**

Is participation in an 8-week asynchronous, app-based personalized coaching intervention informed by biometric data associated with favorable changes in metrics of burnout and well-being among clinicians and other health care professionals?

**Findings:**

In this prospective cohort study of 192 clinicians and other health care professionals who received an 8-week coaching platform intervention, significant score improvements were observed in all domains of the Stanford Professional Fulfillment Index, including burnout, professional fulfillment, and self-valuation.

**Meaning:**

These findings suggest that participation in an 8-week asynchronous, app-based coaching program is associated with lower burnout and higher professional fulfillment and self-valuation among clinicians and other health care professionals.

## Teaser

This cohort study assesses the association between participation in an asynchronous, app-based, personalized coaching intervention informed by biometric data and changes in burnout, professional fulfillment, and self-valuation among health care professionals.

## Introduction

Burnout among health care workers (HCWs) is a widespread occupational syndrome defined by emotional exhaustion, depersonalization, and a diminished sense of personal accomplishment.^1,2,3,4,5^ Rates of burnout surged during the COVID-19 pandemic and remain elevated, posing a sustained threat to HCW well-being, patient care, and the operations of health care systems.^6,7,8,9^ Burnout has been linked to increased medical errors, decreased patient satisfaction, and costly workforce turnover.^10,11,12,13,14^ It is not only a personal experience but also a social phenomenon that spreads through teams and institutions, affecting collective morale, trust, and culture.^8,15,16^

Research has showcased a variety of factors that are associated with burnout, including both system-level and individual-level stressors such as excessive workload, limited psychological safety, reduced autonomy, and impaired sleep.^17,18,19,20,21,22^ The demands of the profession often expose HCWs to chronic stress and sleep deprivation, which can contribute to disruptions in autonomic regulation.^19,23,24,25,26,27^

In response to the growing demands placed on HCWs and the risks posed to health care systems, person-centered interventions have gained increasing traction. Coaching has emerged as a promising person-centered approach to enhance HCW well-being by providing flexible, individualized support that promotes psychological resilience, professional growth, and healthier team dynamics within the complex demands of clinical environments.^28,29,30,31^ Similarly, mobile health solutions have introduced accessible methods for stress management by incorporating mindfulness practices, cognitive behavioral strategies, and stress inoculation techniques.^32,33,34^ These interventions are increasingly recognized for their potential to reduce emotional exhaustion and support adaptive coping in high-pressure settings.^35,36,37,38,39^ While both coaching and mobile health approaches show considerable promise, their broader effectiveness has been limited by the absence of structured feedback loops, inconsistent integration into clinical workflows, and a lack of institutional support that can undermine sustained engagement and long-term impact.^40,41^

This prospective cohort study was designed to examine the implementation and effectiveness of a mobile health intervention designed to meet the evolving needs of active clinicians and other health care professionals.^42^ This approach provided a combination of asynchronous coaching, brief evidence-based learning modules, and biometric feedback to create a flexible, responsive platform to the demands of the HCW environment.^42^ The model integrated brief, evidence-informed practices rooted in performance science with biometric feedback from a wearable photoplethysmography sensor, allowing HCWs to engage with the intervention on their own terms and within their daily constraints. Informed by the Capability, Opportunity, Motivation–Behavior (COM-B) model of behavior change, the intervention described herein aimed to enhance psychological and physical capability through skill-building modules, wearable data, and coaching.^43,44^ We hypothesized that participation in this intervention would be associated with improvements in professional fulfillment, burnout, and self-valuation. Importantly, this approach acknowledges that while many contributors to burnout are systemic, there are also personal levers that HCWs can engage in to support their own recovery, regulation, and sense of agency.

## Methods

### Study Design and Participants

This observational, single-group, pre-to-post cohort study assessed changes in professional fulfillment, burnout, and self-valuation among clinicians and other health care professionals who participated in an 8-week digital coaching program. This study was approved by the Wake Forest University Institutional Review Board, and participants provided electronic informed consent when signing up for the program and logging in to the platform. Participants were able to withdraw consent at any time during the program, and all data would be deleted and excluded from analysis. Participant data were deidentified prior to analysis. Participants were recruited based on convenience sampling from 4 clinical service lines within Advocate Health, including critical care, women’s health, heart and vascular, and emergency medicine, between June 14 and August 12, 2024. Departmental leaders distributed sign-up forms to eligible participants. Inclusion was based on active full-time employment at Advocate Health within those service lines in an HCW role as a clinician or other health care professional (physician, nurse, advanced practice clinician, and technician, among others), possession of an iOS or Android device capable of supporting the digital coaching platform, and absence of substantial comorbidities that could affect the measures of this study. The study followed the Strengthening the Reporting of Observational Studies in Epidemiology (STROBE) reporting guideline.

### Intervention

The intervention consisted of an 8-week asynchronous coaching program delivered through Arena Strive,^42^ a digital coaching platform integrating physiological monitoring with behaviorally anchored coaching, which was conducted between August 27 and October 22, 2024. Participants accessed the program via a mobile app connected to a wearable sensor (2301B Smart Health Ring; Joint Corp) that continuously monitored biometric data, including heart rate variability and bedtime consistency.^26,45^ These data provided real-time feedback to both participants and their coaches.

The program began with a 2-week onboarding period during which participants were mailed a sizing kit for the wearable device, completed demographic intake forms, selected a coach, and had the option to schedule an initial one-on-one call with their coach. Demographic data were collected through self-report via an intake form with which individuals answered questions related to gender, race and ethnicity, primary role, clinical environment, and shift duties. Data on race and ethnicity were collected to provide data transparency in this convenience sampling and account for the intrinsic factor. Participants identified as Black, East Asian, Hispanic or Latino (or Spanish origin), Middle Eastern or North African, Native Hawaiian or Other Pacific Islander, South Asian, White, or other race or ethnicity. Other race or ethnicity encompassed individuals who did not self-identify with the provided categories (responses could include but were not limited to Caribbean, Indigenous, and Southeast or Central Asian). The advanced practice practitioner or clinician role was internally defined as nurse practitioners and physician assistants, but ultimately participants self-identified their roles. After onboarding, participants received their wearable sensor, began syncing biometric data, and gained access to the full digital coaching platform experience. eAppendix 1 in Supplement 1 presents a detailed participant and coaching intervention timeline.

The core curriculum of the program was structured around 3 primary performance domains, which included regulating stress, promoting recovery, and stabilizing energy. Participants navigated a self-guided video content journey designed to be completed in less than 5 minutes per session (eAppendix 2 in Supplement 1). The content was intentionally designed to be short in format to accommodate the demands of active clinicians and other health care professionals and improve engagement.

Coaching interactions occurred asynchronously through a secure chat interface. Participants were encouraged to engage with their coach an average of 3 times per week to reflect on physiologic trends, set personalized goals, and apply evidence-informed tools. Two optional synchronous one-on-one calls with their coach, one at the program’s start and one at the conclusion, were also available. The coaching model emphasized autonomy, personalization, and habit formation, using biometric data to support real-time behavior change.

### Outcome Measures

Psychological measures were evaluated using self-administered surveys, the Stanford Professional Fulfillment Index (PFI)^46,47^ and the Maslach Burnout Inventory Human Services Survey (MBI-HSS),^5,48^ collected at the beginning (week 0) and end (week 8) of the program. Surveys were completed within the digital coaching platform through a Typeform survey (Typeform Inc) and involved self-reported responses.

The PFI evaluates professional fulfillment, burnout, and self-valuation.^46,47^ Burnout reflects work-related exhaustion and interpersonal disengagement, while professional fulfillment denotes the intrinsic reward an individual derives from their work.^46^ Self-valuation describes an individual’s capacity to prioritize personal well-being in a constructive, compassionate manner under professional demands and setbacks. In physicians, self-valuation correlates with both burnout and sleep-related impairment.^47^ The professional fulfillment domain includes 6 items scored on a 5-point Likert scale ranging from 0 (not at all true) to 4 (completely true). The burnout domain includes 10 items scored from 0 (not at all) to 4 (extremely). Domain scores for professional fulfillment and burnout are calculated as the mean of their respective items and range from 0 to 4. According to previous validation work, a mean burnout score of 1.33 or higher signifies a positive burnout screen, while a mean professional fulfillment score of 3.00 or greater corresponds to an individual reporting a “very good” quality of life.^46^ The self-valuation domain consists of 4 items from 0 (never) to 4 (always) and is summed to yield a total score between 0 and 16. Scores of 8 or less correspond to an increased burnout risk.^47^

The MBI-HSS assesses emotional exhaustion (feelings of being emotionally overextended and exhausted by one’s work), depersonalization (an unfeeling and impersonal response toward recipients of one’s service, care treatment, or instruction), and personal accomplishment (feelings of competence and successful achievement in one’s work).^48^ Respondents rate 22 statements on a 7-point frequency scale from 0 (never) to 6 (every day). The emotional exhaustion subscale comprises 9 items summed to yield a score between 0 and 54.^48^ Scores of 27 or higher indicate high exhaustion, scores from 17 to 26 indicate moderate exhaustion, and scores of 16 or less indicate low exhaustion. The depersonalization subscale comprises 5 items summed to yield a score between 0 and 30. Scores of 13 or higher indicate high depersonalization, scores from 7 to 12 indicate moderate depersonalization, and scores of 6 or less indicate low depersonalization. The personal accomplishment subscale comprises 8 items summed to yield a score between 0 and 48. Scores of 31 or less indicate low personal accomplishment, scores from 32 to 38 indicate moderate personal accomplishment, and scores of 39 or higher indicate high personal accomplishment.^48^

### Statistical Analysis

All statistical analyses were performed using Python, version 3.8, and the statsmodels package, version 0.14.4 (Python Software Foundation). For each outcome, linear mixed-effects models were used with restricted maximum likelihood estimation. Fixed effects were specified for time (week 0 vs week 8), and random intercepts were included to account for repeated measures within individuals. Analyses were conducted using data from participants with complete pairs of observations, defined as individuals who completed both baseline (week 0) and follow-up (week 8) assessments for the same outcome measure.

To address multiplicity across the 6 prespecified primary outcomes (PFI professional fulfillment, burnout, and self-valuation and MBI-HSS emotional exhaustion, depersonalization, and personal accomplishment), we applied a Bonferroni correction with a 2-sided significance threshold of α = .008 (.05/6). Missing data were assumed to be missing at random. Diagnostic plots (residuals vs fitted values and quantile-quantile plots) were used to assess model assumptions, including normality and homoscedasticity. Statistical significance was defined as 2-sided *P* < .05. Analysis was completed in November 2024 and April 2025.

## Results

**Figure 1.**
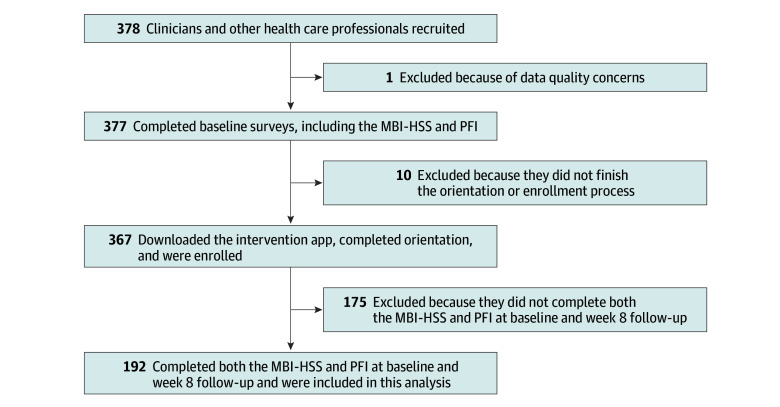
Study Flow Diagram MBI-HSS indicates Maslach Burnout Inventory Human Services Survey; PFI, Stanford Professional Fulfillment Index.

Over a 2-month recruitment window, 367 clinicians and other health care professionals downloaded the asynchronous mobile app, completed an intake form and orientation videos, and enrolled in the program. Electronic surveys, including the PFI and the MBI-HSS, were delivered at baseline (week 0) and follow-up (week 8). Of the 367 individuals enrolled, the 192 who completed both the PFI and MBI-HSS at both time points were included in this study, yielding an overall paired-survey response rate of 52.3%. Participant flow and subsequent analysis is presented in Figure 1. The analytic sample was predominantly female (135 [70.3%] compared with 57 male participants [29.7%]), with a mean (SD) age of 42.5 (10.0) years. Participants identified as Black (10 [5.2%]), East Asian (8 [4.2%]), Hispanic, Latino, or Spanish origin (3 [1.6%]), Middle Eastern or North African (4 [2.1%]), Native Hawaiian or Other Pacific Islander (1 [0.5%]), South Asian (13 [6.8%]), White (145 [75.5%]), or other race or ethnicity (8 [4.2%]). The study population was primarily composed of attending physicians (92 [47.9%]), advanced practice clinicians (eg, nurse practitioner, physician assistant; 47 [24.5%]), and nurses (37 [19.3%]). Emergency medicine (78 [40.6%]) and women’s health (54 [28.1%]) were the most common specialties, and most participants worked day shifts (133 [69.3%]). Other demographic and professional characteristics are detailed in Table 1.

**Table 1.** Participant Demographic and Professional Characteristics

Characteristic	No. (%) of participants (N = 192)
Gender	
Female	135 (70.3)
Male	57 (29.7)
Race and ethnicity	
Black	10 (5.2)
East Asian	8 (4.2)
Hispanic, Latino, or Spanish origin	3 (1.6)
Middle Eastern or North African	4 (2.1)
Native Hawaiian or Other Pacific Islander	1 (0.5)
South Asian	13 (6.8)
White	145 (75.5)
Other race or ethnicity^a^	8 (4.2)
Primary role	
Attending physician	92 (47.9)
Advanced practice clinician (eg, nurse practitioner, physician assistant)	47 (24.5)
Nurse	37 (19.3)
Administrator	6 (3.1)
Trainee	5 (2.6)
Resident physician	1 (0.5)
Health care technician	1 (0.5)
Nurse manager, education, or emergency department	1 (0.5)
Registered nurse clinical supervisor	1 (0.5)
Other advanced clinician	1 (0.5)
Scope of medicine	
Emergency medicine	78 (40.6)
Women’s health	54 (28.1)
Heart and vascular	30 (15.6)
Critical care	28 (14.6)
Other	2 (1.0)
Clinical environment	
Emergency department	78 (40.6)
Inpatient units	49 (25.5)
Outpatient clinical area	41 (21.4)
Other procedural area	9 (4.7)
Other	9 (4.7)
Operating rooms	6 (3.1)
Shift time	
Days	133 (69.3)
Nights	30 (15.6)
Evenings	29 (15.1)

^a^ Encompasses individuals who did not self-identify with the provided categories (responses could include but were not limited to Caribbean, Indigenous, and Southeast or Central Asian).

Participants completed a mean (SD) of 13.6 (7.6) lessons and sent a mean (SD) of 19.6 (69.5) messages to their coach. Introductory calls were scheduled by 177 participants (92.2%), and wrap-up calls were scheduled by 110 (57.3%).

### PFI Domains

**Figure 2.**
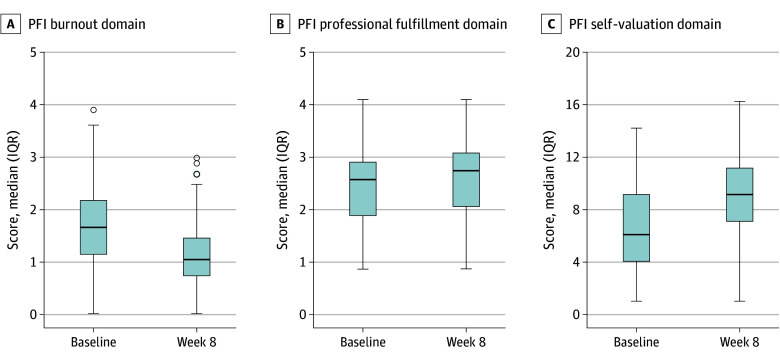
Reported Distribution of Stanford Professional Fulfillment Index (PFI) Subscale Scores at Baseline and Week 8 A to C, Box-and-whisker plots for the PFI burnout (A), professional fulfillment (B), and self-valuation (C) domains at baseline and week 8. Box plots display the distribution of PFI burnout scores at baseline (week 0) and follow-up (week 8). The center line within each box represents the median score, box boundaries indicate the IQR (25th to 75th percentiles), and whiskers extend to 1.5 times the IQR. Individual circles represent outliers beyond the whisker limits. Statistically significant changes from baseline to week 8 (*P* < .05) were observed for all 3 domains.

Statistically significant improvements in scores on the PFI domains from baseline to week 8 were observed (Figure 2 and Table 2). The mean (SD) total burnout score declined from 1.60 (0.71) to 1.09 (0.62) (change, −0.51 [95% CI, −0.60 to −0.43] points; *P* < .001), while the mean (SD) self-valuation score increased from 6.47 (3.00) to 9.02 (2.75) (change, 2.55 [95% CI, 2.12 to 2.98] points; *P* < .001) and the mean (SD) professional fulfillment score rose from 2.36 (0.68) to 2.56 (0.76) (change, 0.20 [95% CI, 0.12 to 0.28] points; *P* < .001).

**Table 2.** Changes in PFI Domain Scores From Baseline to Week 8

PFI domain	Score, mean (SD), points		Change (95% CI), points	Unadjusted *P* value
	Week 0	Week 8		
**Professional fulfillment**				
Total score	2.36 (0.68)	2.56 (0.76)	0.20 (0.12 to 0.28)	<.001
Item				
I feel happy at work.	1.97 (0.85)	2.19 (0.96)	0.22 (0.09 to 0.35)	.001
I feel worthwhile at work.	2.41 (0.98)	2.56 (0.99)	0.15 (0.03 to 0.28)	.02
My work is satisfying to me.	2.36 (0.92)	2.61 (0.91)	0.25 (0.13 to 0.37)	<.001
I feel in control when dealing with difficult problems at work.	1.98 (0.92)	2.44 (0.89)	0.46 (0.32 to 0.59)	<.001
My work is meaningful to me.	2.93 (0.86)	2.92 (0.91)	−0.01 (−0.12 to 0.11)	.93
I’m contributing professionally (eg, patient care, teaching, research, and leadership) in the ways I value most.	2.51 (0.94)	2.65 (0.90)	0.14 (0.02 to 0.27)	.03
**Burnout**				
Total score	1.60 (0.71)	1.09 (0.62)	−0.51 (−0.60 to −0.43)	<.001
Item response to this statement: “During the past 2 weeks, I have felt…”				
A sense of dread when I think about the work I have to do	1.79 (0.96)	1.31 (0.85)	−0.48 (−0.61 to −0.35)	<.001
Physically exhausted at work	2.02 (0.94)	1.59 (0.85)	−0.43 (−0.57 to −0.29)	
Lacking in enthusiasm at work	1.73 (0.88)	1.32 (0.83)	−0.41 (−0.54 to −0.29)	
Emotionally exhausted at work	2.09 (1.04)	1.53 (0.98)	−0.56 (−0.69 to −0.42)	
Item response to this statement: “During the past 2 weeks, my job has contributed to me feeling…”				
Less empathetic with my patients	1.41 (0.98)	0.97 (0.84)	−0.44 (−0.56 to −0.32)	<.001
Less empathetic with my colleagues	1.49 (0.99)	0.86 (0.80)	−0.63 (−0.76 to −0.50)	
Less sensitive to others’ feelings/emotions	1.43 (0.91)	0.86 (0.75)	−0.57 (−0.69 to −0.45)	
Less interested in talking with my patients	1.34 (0.99)	0.85 (0.84)	−0.49 (−0.60 to −0.36)	
Less connected with my patients	1.35 (0.96)	0.81 (0.77)	−0.54 (−0.67 to −0.43)	
Less connected with my colleagues	1.37 (1.01)	0.80 (0.73)	−0.57 (−0.71 to −0.43)	
**Self-valuation**				
Total score	6.47 (3.00)	9.02 (2.75)	2.55 (2.12 to 2.98)	<.001
Item				
When I made a mistake, I felt more self-condemnation than self-encouragement to learn from the experience.	1.81 (1.04)	2.47 (0.88)	0.66 (0.49 to 0.82)	<.001
I was less compassionate with myself than I was with others.	1.42 (1.04)	2.10 (0.94)	0.68 (0.53 to 0.83)	
I put off taking care of my own health due to time pressure.	1.33 (0.89)	2.02 (0.92)	0.69 (0.55 to 0.82)	
Taking care of my needs seemed incompatible with taking care of my patients’ needs.	1.91 (0.95)	2.43 (0.87)	0.52 (0.38 to 0.66)	

Abbreviation: PFI, Stanford Professional Fulfillment Index.

The mean (SD) score for the item “I feel in control when dealing with difficult problems at work” had the largest change, with an increase from 1.98 (0.92) to 2.44 (0.89) (change, 0.46 [95% CI, 0.32 to 0.59] points; *P* < .001). In contrast, scores on other PFI items such as feeling worthwhile at work and contributing meaningfully showed minimal change (Table 2). Moreover, the mean (SD) score for the item “My work is meaningful to me” actually decreased from 2.93 (0.86) to 2.92 (0.91) (change, −0.01 [95% CI, −0.12 to 0.11] points; *P* = .93).

### MBI-HSS Subscales

Statistically significant changes in scores on the MBI-HSS subscales were similarly observed from baseline to week 8 (Table 3). The mean (SD) emotional exhaustion score decreased from 19.74 (8.95) to 15.68 (8.59) (change, −4.06 [95% CI, −5.10 to −3.03] points; *P* < .001), the mean (SD) depersonalization score decreased from 14.58 (8.50) to 10.32 (7.19) (change, −4.26 [95% CI, −5.26 to −3.27] points; *P* < .001), and the mean (SD) personal accomplishment score increased from 37.68 (6.85) to 39.19 (6.80) (change, 1.51 [95% CI, 0.75 to 2.28] points; *P* < .001).

**Table 3.** Changes in MBI-HSS Subscale Scores From Baseline to Week 8

MBI-HSS subscale	Score, mean (SD), points		Change (95% CI), points	Unadjusted *P* value
	Week 0	Week 8		
**Emotional exhaustion**				
Total score (sum of items 1-9)	19.74 (8.95)	15.68 (8.59)	−4.06 (−5.10 to −3.03)	<.001
Item				
1	3.73 (1.50)	2.89 (1.49)	−0.84 (1.05 to −0.63)	<.001
2	3.76 (1.72)	3.09 (1.82)	−0.67 (−0.91 to −0.42)	<.001
3	3.36 (1.73)	2.51 (1.69)	−0.85 (−1.09 to −0.62)	<.001
4	1.37 (1.57)	1.17 (1.35)	−0.20 (−0.41 to 0)	.06
5	2.80 (1.76)	2.20 (1.57)	−0.60 (−0.80 to −0.39)	<.001
6	3.31 (1.64)	2.62 (1.53)	−0.69 (−0.91 to −0.47)	<.001
7	3.48 (1.67)	2.75 (1.73)	−0.73 (−0.95 to −0.51)	<.001
8	1.37 (1.57)	1.17 (1.35)	−0.20 (−0.41 to 0)	.06
9	1.30 (1.60)	0.95 (1.27)	−0.35 (−0.53 to −0.16)	<.001
**Depersonalization**				
Total score (sum of items 10-14)	14.58 (8.50)	10.32 (7.19)	−4.26 (−5.26 to −3.27)	<.001
Item				
10	1.29 (1.62)	0.90 (1.26)	−0.39 (−0.60 to −0.19)	<.001
11	1.99 (1.75)	1.22 (1.40)	−0.77 (−0.99 to −0.56)	<.001
12	1.73 (1.84)	1.15 (1.48)	−0.58 (−0.79 to −0.38)	<.001
13	0.64 (1.15)	0.50 (0.99)	−0.14 (−0.31 to 0.03)	.10
14	2.73 (1.82)	1.99 (1.72)	−0.74 (−1.01 to −0.46)	<.001
**Personal accomplishment**				
Total score (sum of items 15-22)	37.68 (6.85)	39.19 (6.80)	1.51 (0.75 to 2.28)	<.001
Item				
15	4.88 (1.29)	4.91 (1.22)	0.03 (−0.16 to 0.22)	.79
16	5.30 (0.97)	5.36 (1.03)	0.06 (−0.07 to 0.20)	.36
17	5.07 (1.13)	5.27 (1.01)	0.20 (0.05 to 0.35)	.008
18	3.53 (1.65)	4.10 (1.42)	0.57 (0.38 to 0.77)	<.001
19	5.24 (1.05)	5.30 (0.99)	0.06 (−0.08 to 0.20)	.42
20	3.94 (1.79)	4.34 (1.67)	0.40 (0.19 to 0.62)	<.001
21	4.49 (1.37)	4.58 (1.47)	0.09 (−0.11 to 0.30)	.37
22	5.24 (0.93)	5.33 (0.89)	0.09 (−0.06 to 0.25)	.22

Abbreviation: MBI-HSS, Maslach Burnout Inventory Human Services Survey.

We report unadjusted *P* values from the primary models in Table 2 and Table 3. Conclusions were unchanged after correction, with associations remaining between the intervention and changes in all 6 primary outcomes (PFI domains and MBI-HSS subscales) between baseline and week 8.

## Discussion

In this study, participation in an 8-week asynchronous, app-based personalized coaching intervention informed by the participant’s biometric data was associated with improvement in professional fulfillment, burnout, and self-valuation among clinicians and other health care professionals across multiple service lines of a large health care system. These outcomes were observed across 2 validated instruments: the PFI and the MBI-HSS.

This study contributes observational evidence that such improvements can be achieved in association with an asynchronous, technology-enabled model embedded into daily routines, although causal conclusions cannot be drawn from this design. These findings align with and extend prior work on coaching and mobile health interventions in clinical populations. Coaching has been shown to reduce emotional exhaustion and improve adaptive coping among physicians and nurses.^28,29,39^ After completion of the 8-week program, the mean emotional exhaustion score, as measured by the MBI-HSS, decreased by −4.06 (95% CI, −5.10 to −3.03) points (*P* < .001). The WISER trial by Profit et al,^38^ which delivered 10 “bite-size” positive-psychology exercises in periods of 10 days, reported a significant decrease of −5.64 (95% CI, −9.59 to −1.68) points (*P* = .005) in the emotional exhaustion score in 1 month, using a derivative of the MBI emotional exhaustion subscale. This close alignment of intervention outcomes supports that brief, digitally delivered intervention can be associated with a timely and meaningful reduction in validated measures of emotional exhaustion reduction among clinicians and other health care professionals.

The asynchronous platform reported herein was deliberately designed using the COM-B framework to overcome common implementation and usage barriers such as time constraints, scheduling rigidity, and lack of contextual relevance by providing capability through readily available concise and targeted tools (eAppendix 2 in Supplement 1).^43^ Participation and engagement in this program were high. Over the 8-week asynchronous coaching program, the mean (SD) professional fulfillment score on the PFI increased by 0.20 (95% CI, 0.12 to 0.28) points (*P* < .001), with the largest change seen in the item that assessed feeling in control at work when dealing with difficult problems. Similar to these findings, the Stanford IMPACT trial, which delivered coaching through 4 biweekly workshops led by opinion leaders at the clinic level, resulted in increases in professional fulfillment score (0.63 points; *P* = .001) over a 1-year period.^39^ Although the peer-led intervention resulted in greater increases in professional fulfillment, a greater reduction in burnout was seen with the 8-week asynchronous coaching intervention. These findings suggest that asynchronous, data-driven microlearning and coaching may be particularly useful for—and were associated with—greater reductions in emotional exhaustion, whereas peer-led workshops and group coaching may have a greater effect on professional fulfillment.

In contrast with the PFI results related to feeling in control at work, scores on other PFI items such as feeling worthwhile at work and contributing meaningfully showed minimal change. Moreover, the mean score on the item feeling the work is meaningful actually decreased. These unanticipated results present opportunities to enhance modules focused on mission alignment and emotional resilience—or perhaps suggest that these domains are refractory to this or similar interventions. Further research in this area is needed.

While biometric data such as heart rate variability and bedtime consistency were not analyzed in this study, they were central to the program design. These data provided participants with daily real-time physiological feedback and served as anchors for the coaching conversations. Although these data were not required for participation, this data stream enabled individualized goal setting and reinforced behavioral insights. Future research should assess whether early shifts in these markers predict downstream psychological improvement and whether examining baseline biometric profiles alongside survey scores could enable early identification of those most at risk, allowing organizational leaders to offer more timely support. The role of biometric trends as predictive, privacy-preserving early indicators of burnout remains an especially promising area for system-level learning.

### Limitations

This study has several limitations that should be acknowledged. First, this was a single-group, pre-to-post cohort study conducted without a concurrent control group; therefore, causal inference is limited, and observed changes may reflect factors other than the intervention, including regression to the mean, Hawthorne and expectancy effects, effects of repeated measurement, and secular changes in workload, staffing, or institutional initiatives during the study period. Regression to the mean is possible; however, baseline well-being levels were not uniformly extreme across domains, suggesting regression to the mean alone may not fully account for the pattern of changes observed. Participation was self-selected, and analyses were restricted to individuals who completed both baseline and week 8 assessments, which may introduce selection bias. Although the follow-up completion rate was 52.3%, we compared completers with noncompleters and found no significant differences in available demographics or baseline PFI or MBI-HSS scores, supporting the missing-at-random assumption; however, unmeasured differences may still contribute to attrition bias. We chose to not perform multiple imputation because only a limited set of auxiliary variables beyond the outcomes themselves were available to inform an imputation model, and imputing the follow-up values under these conditions would rely on strong, untestable assumptions about the missingness mechanism. Given the lack of baseline differences between completers and noncompleters, we judged a complete-case analysis with transparent reporting of attrition to be more appropriate than model-based imputation that might introduce additional bias.

Second, the multimodal nature of the platform used in this study prevents isolation of the relative contributions of coaching, microlearning content, and biometric feedback. However, exploratory analyses showed no evidence of a linear dose-response association between the number of messages sent to a coach and changes in the primary outcomes, suggesting that factors other than message volume (eg, content quality or level of biometric awareness) may be important. Finally, we did not assess prior wearable use or familiarity with commercial wearable biometrics, which could alone influence the perception or impact of biometric-informed feedback and coaching. Given the multimodal platform, we could not isolate the association with the wearable, coaching, and content-viewing. Future research involving multifactorial designs would help to isolate these outcomes. Because the intervention was implemented within a single health care system, generalizability may be limited, as organizational context and support for clinician and other health care professional well-being vary across institutions.

## Conclusions

In this cohort study of active clinicians and other health care professionals, participation in an 8-week asynchronous app-based coaching program supported with biometric data was associated with reductions in burnout and improvements in professional fulfillment and self-valuation. These findings suggest that a flexible biometric-informed coaching model can be feasibly integrated into demanding clinical workflows to support HCW well-being. Although the absence of a control group limits causal inference, the medium to large effect sizes observed across validated measures underscore the potential value of digital coaching platforms and highlight the need for clinical trials to test whether these approaches can mitigate the personal challenges inherent to the HCW environment. Future research should examine the association between physiological markers and self-reported outcomes and evaluate whether these signals can serve as passive objective measures of declining well-being among clinicians and other health care professionals.

## Notes

Supplement 1. **eAppendix 1.** Coaching Participant Intervention Timeline **eAppendix 2.** Asynchronous Coaching Description
eReferences.

Supplement 2.
Data Sharing Statement
