# Supplementary material for: Assessment of Clinician Well-Being Using a Biometric-Informed Coaching Platform
Source: JAMA Netw Open. 2026 Feb 11;9(2):e2558865. doi: 10.1001/jamanetworkopen.2025.58865 (PMC12895280; doi:10.1001/jamanetworkopen.2025.58865)
Supplement: Supplement 1. — eAppendix 1. Coaching Participant Intervention Timeline eAppendix 2. Asynchronous Coaching Description eReferences. [file jamanetwopen-e2558865-s001.pdf]

Supplemental Online Content

Leo T, Reynolds J, Blair J, et al. Assessment of clinician well-being using a biometric-informed coaching platform. *JAMA Netw Open*. 2026;9(2):e2558865. doi:10.1001/jamanetworkopen.2025.58865

eAppendix 1. Coaching Participant Intervention Timeline

eAppendix 2. Asynchronous Coaching Description

eReferences.

This supplemental material has been provided by the authors to give readers additional information about their work.

© 2026 Leo T et al. *JAMA Network Open*.

eAppendix 1. Coaching Participant Intervention Timeline

Activity	Week 0	Week 1	Week 2	Week 3	Week 4	Week 5	Week 6	Week 7
**Demographic form**	X							
**Week 0 PFI & MBI**	X							
**Ring sensor sizing kit shipped**	X							
**Ring size entered, wearable shipped**	X	X						
**Coach selection via app**	X	X	X					
**Optional Coach onboarding call**			X					
**Weekly content modules**			X	X	X	X	X	X
**Asynchronous coaching (approximate 3 coaching messages a week)**			X	X	X	X	X	X
**Biometric syncing (HRV, bedtime)**			X	X	X	X	X	X
**Optional Coach Wrap-up call**								X
**Week 8 PFI & MBI**								X

© 2026 Leo T et al. *JAMA Network Open*.

eAppendix 2. Asynchronous Coaching Description

Intervention Overview

Arena Strive is a digital, app-based intervention designed to reduce burnout and enhance recovery among frontline healthcare professionals. Grounded in performance science, the program incorporates brief, evidence-informed practices across psychological and physiological domains. It is structured for low-burden use in high-demand clinical environments.

The Arena Strive approach is informed by the COM-B model of behavior change, which emphasizes three essential conditions for sustained behavior adoption: Capability, Opportunity, and Motivation[1]. These principles are embedded across the platform's design:

- Capability is enhanced through micro-learning modules that build skills in stress regulation, recovery, and energy management.
- Opportunity is provided through seamless integration of tools into the clinical workflows, with on-demand access to personalized coaching and biometric feedback.
- Motivation is supported by continuous feedback loops, coach nudges, and self-monitoring features that reinforce progress and intrinsic growth.

The app contains three main tabs:

1. Content Pillars: A curated library of self-guided micro-learning modules.
2. Asynchronous Coach Chat: Asynchronous messaging with certified performance coaches for individualized support.
3. Data Dashboard: Displays physiological data from the J-Style biometric ring, including Heart Rate Variability (HRV) and bedtime consistency, as markers of recovery and adaptation.

All video modules are under 5 minutes in length and paired with an optional short-form practice (2–5 minutes), promoting engagement without disrupting clinical schedules.

The Arena Strive content is structured across four core performance domains, each addressing specific psychological or behavioral challenges with targeted tools:

Performance Pillar	Tool	Description
**Regulate Stress**	Activation Breath [2]	Brief breath practice to raise alertness before tasks.
	Visualization [3]	Mental rehearsal technique to prepare for high-stakes events.
	Energizing Movement [4]	Short physical activity to increase daily energy levels.
	Relaxation Movement [5]	Gentle stretches to reduce tension and promote calm.
	Panoramic Vision [6]	Visual reset to downshift stress in acute moments.
	Deactivation Breath [7]	Simple breath technique to quickly reduce arousal.

© 2026 Leo T et al. *JAMA Network Open*.

	Situational Dialogue [8]	Guided reflection for navigating difficult conversations.
	After Action Review [9]	Structured debrief to process and learn from events.
	Stimulus Labeling	Cognitive tool to name and reframe emotional stressors.
**Promote Regeneration**[10]	Cold, Dark, Quiet [11]	Environmental sleep hygiene checklist to improve rest.
	Light Restriction [12]	Evening light control to support circadian rhythm.
	Sleep Scheduling [13]	Behavioral cueing to anchor bedtime routines.
	Yoga Nidra [14]	Deep rest script to recharge in short windows.
	Self-Hypnosis [15]	Audio-based script to facilitate sleep onset.
	Box Breath [16]	Rhythmic breathing to refocus or unwind.
	Active Recovery [17]	Low-intensity movement to aid physical and mental recovery.
	Examine Gratitude [18,19]	Reflective writing to build positive emotional state.
	Debriefing [20]	Brief solo or team reflection to synthesize experiences.
**Stabilize Energy**	AM/PM Routine [21]	Short routine builder to create consistent bookends to the day.
	Minimum Effective Dose[22]	Quick workout designed for high return, low fatigue.
	Exercise Snacks [23]	Micro-activities to maintain physical activation.
	Mind Diet [24]	Food guidance focused on cognitive and cardiovascular health.
	Energy Stabilization [25]	Dietary choices to prevent sugar crashes and energy dips.
	Strategic Food Timing [26]	Timing meals to optimize metabolism and performance.
	Shift Sleep/Wake [13]	Behavioral resets for rotating between shifts.
	Exercise Timing [27]	Aligning workouts with natural circadian energy peaks.
	Fuel & Supplements [28,29]	Education on nutrition and supplements for sustained energy

Coaching engagement strategy:

Participants had continuous access to the Arena Strive platform and were encouraged to engage daily with both self-guided content and biometric feedback. Users could message their assigned performance coach

© 2026 Leo T et al. *JAMA Network Open*.

at any time via the asynchronous in-app chat. Coaches were expected to reply within 24 hours and typically initiated contact every three days.

In addition to on-demand communication, coaches were required to send three structured messages per week, personalized based on patterns observed in biometric data (e.g., HRV trends, sleep regularity) and engagement with video modules.

These touchpoints serve to reinforce learning, provide contextual support, and suggest next steps based on individual performance and recovery trends. This hybrid of user-initiated and coach-initiated contact was designed to balance personalization with scalability in high-demand clinical environments.

© 2026 Leo T et al. *JAMA Network Open*.

eReferences.

1. Willmott TJ, Pang B, Rundle-Thiele S. Capability, opportunity, and motivation: an across contexts empirical examination of the COM-B model. *BMC Public Health*. 2021;21(1):1014. doi:10.1186/s12889-021-11019-w

2. Bordoni B, Purgol S, Bizzarri A, Modica M, Morabito B. The Influence of Breathing on the Central Nervous System. *Cureus*. 10(6):e2724. doi:10.7759/cureus.2724

3. Schuster C, Hilfiker R, Amft O, et al. Best practice for motor imagery: a systematic literature review on motor imagery training elements in five different disciplines. *BMC Med*. 2011;9(1):75. doi:10.1186/1741-7015-9-75

4. Füzéki E, Engeroff T, Banzer W. Health Benefits of Light-Intensity Physical Activity: A Systematic Review of Accelerometer Data of the National Health and Nutrition Examination Survey (NHANES). *Sports Med Auckl NZ*. 2017;47(9):1769-1793. doi:10.1007/s40279-017-0724-0

5. Toussaint L, Nguyen QA, Roettger C, et al. Effectiveness of Progressive Muscle Relaxation, Deep Breathing, and Guided Imagery in Promoting Psychological and Physiological States of Relaxation. *Evid-Based Complement Altern Med ECAM*. 2021;2021:5924040. doi:10.1155/2021/5924040

6. Marois A, Charbonneau B, Szolosi AM, Watson JM. The Differential Impact of Mystery in Nature on Attention: An Oculometric Study. *Front Psychol*. 2021;12:759616. doi:10.3389/fpsyg.2021.759616

7. Chaitanya S, Datta A, Bhandari B, Sharma VK. Effect of Resonance Breathing on Heart Rate Variability and Cognitive Functions in Young Adults: A Randomised Controlled Study. *Cureus*. 14(2):e22187. doi:10.7759/cureus.22187

8. Overton AR, Lowry AC. Conflict Management: Difficult Conversations with Difficult People. *Clin Colon Rectal Surg*. 2013;26(4):259-264. doi:10.1055/s-0033-1356728

9. After-Action Reviews: A Simple Yet Powerful Tool. Wharton Executive Education. Accessed July 15, 2025. https://executiveeducation.wharton.upenn.edu/thought-leadership/wharton-at-work/2021/07/after-action-reviews-simple-tool/

10. Frontiers | The Temperature Dependence of Sleep. Accessed July 15, 2025. https://www.frontiersin.org/journals/neuroscience/articles/10.3389/fnins.2019.00336/full

11. Tähkämö L, Partonen T, Pesonen AK. Systematic review of light exposure impact on human circadian rhythm. *Chronobiol Int*. 2019;36(2):151-170. doi:10.1080/07420528.2018.1527773

12. Navara KJ, Nelson RJ. The dark side of light at night: physiological, epidemiological, and ecological consequences. *J Pineal Res*. 2007;43(3):215-224. doi:10.1111/j.1600-079X.2007.00473.x

13. Sletten TL, Weaver MD, Foster RG, et al. The importance of sleep regularity: a consensus statement of the National Sleep Foundation sleep timing and variability panel. *Sleep Health J Natl Sleep Found*. 2023;9(6):801-820. doi:10.1016/j.sleh.2023.07.016

14. Amita S, Prabhakar S, Manoj I, Harminder S, Pavan T. Effect of yoga-nidra on blood glucose level in diabetic patients. *Indian J Physiol Pharmacol*. 2009;53(1):97-101.

© 2026 Leo T et al. *JAMA Network Open*.

15. Snyder M, Alldredge CT, Stork SR, Elkins GR. Feasibility of a Self-Administered Hypnosis Intervention for Improving Sleep in College Students. *Int J Clin Exp Hypn*. 2023;71(4):297-312. doi:10.1080/00207144.2023.2249047

16. Balban MY, Neri E, Kogon MM, et al. Brief structured respiration practices enhance mood and reduce physiological arousal. *Cell Rep Med*. 2023;4(1):100895. doi:10.1016/j.xcrm.2022.100895

17. Dupuy O, Douzi W, Theurot D, Bosquet L, Dugué B. An Evidence-Based Approach for Choosing Post-exercise Recovery Techniques to Reduce Markers of Muscle Damage, Soreness, Fatigue, and Inflammation: A Systematic Review With Meta-Analysis. *Front Physiol*. 2018;9:403. doi:10.3389/fphys.2018.00403

18. Kaczmarek LD, Kashdan TB, Drążkowski D, et al. Why do people prefer gratitude journaling over gratitude letters? The influence of individual differences in motivation and personality on web-based interventions. *Personal Individ Differ*. 2015;75:1-6. doi:10.1016/j.paid.2014.11.004

19. Gratitude | SpringerLink. Accessed July 15, 2025. https://link.springer.com/rwe/10.1007/978-94-007-0753-5_3313

20. Do Team and Individual Debriefs Enhance Performance? A Meta-Analysis - Scott I. Tannenbaum, Christopher P. Cerasoli, 2013. Accessed July 15, 2025. https://journals.sagepub.com/doi/abs/10.1177/0018720812448394

21. Arlinghaus KR, Johnston CA. The Importance of Creating Habits and Routine. *Am J Lifestyle Med*. 2018;13(2):142-144. doi:10.1177/1559827618818044

22. Wender CLA, Manninen M, O'Connor PJ. The Effect of Chronic Exercise on Energy and Fatigue States: A Systematic Review and Meta-Analysis of Randomized Trials. *Front Psychol*. 2022;13:907637. doi:10.3389/fpsyg.2022.907637

23. Jones MD, Clifford BK, Stamatakis E, Gibbs MT. Exercise Snacks and Other Forms of Intermittent Physical Activity for Improving Health in Adults and Older Adults: A Scoping Review of Epidemiological, Experimental and Qualitative Studies. *Sports Med Auckl NZ*. 2024;54(4):813-835. doi:10.1007/s40279-023-01983-1

24. Morris MC, Tangney CC, Wang Y, et al. MIND diet slows cognitive decline with aging. *Alzheimers Dement J Alzheimers Assoc*. 2015;11(9):1015-1022. doi:10.1016/j.jalz.2015.04.011

25. Mantantzis K, Schlaghecken F, Sünram-Lea SI, Maylor EA. Sugar rush or sugar crash? A meta-analysis of carbohydrate effects on mood. *Neurosci Biobehav Rev*. 2019;101:45-67. doi:10.1016/j.neubiorev.2019.03.016

26. Timing of meals: when is as critical as what and how much - PubMed. Accessed July 15, 2025. https://pubmed.ncbi.nlm.nih.gov/28143856/

27. Puetz TW. Physical activity and feelings of energy and fatigue: epidemiological evidence. *Sports Med Auckl NZ*. 2006;36(9):767-780. doi:10.2165/00007256-200636090-00004

28. Saghafian F, Hajishafiee M, Rouhani P, Saneei P. Dietary fiber intake, depression, and anxiety: a systematic review and meta-analysis of epidemiologic studies. *Nutr Neurosci*. 2023;26(2):108-126. doi:10.1080/1028415X.2021.2020403

© 2026 Leo T et al. *JAMA Network Open*.

29. Gannon MC, Nuttall FQ, Saeed A, Jordan K, Hoover H. An increase in dietary protein improves the blood glucose response in persons with type 2 diabetes12. *Am J Clin Nutr*. 2003;78(4):734-741. doi:10.1093/ajcn/78.4.734

© 2026 Leo T et al. *JAMA Network Open*.
