# Supplementary material for: Assessment of Clinician Well-Being Using a Biometric-Informed Coaching Platform
Source: JAMA Netw Open. 2026 Feb 11;9(2):e2558865. doi: 10.1001/jamanetworkopen.2025.58865 (PMC12895280; doi:10.1001/jamanetworkopen.2025.58865)
Supplement: Supplement 2. — Data Sharing Statement [file jamanetwopen-e2558865-s002.pdf]

Data Sharing Statement

Leo. Assessment of Clinician Well-Being Using a Biometric-Informed Coaching Platform. *JAMA Netw Open*. Published February 11, 2026. doi:10.1001/jamanetworkopen.2025.58865

Data

Data available: Individual participant data will not be shared because the dataset includes sensitive information from a limited group of health care professionals and is subject to data use agreements with a commercial partner (Arena Labs) that preclude public data sharing.
